# Therapeutic Immunomodulation in Cardiovascular Disease: Anti-inflammatory Strategies and Landmark Trials

**DOI:** 10.7759/cureus.99835

**Published:** 2025-12-22

**Authors:** Muhammad Awais, Mohanad Baroudi, Muhammad Hasnain Mankani, Aizaz Ali, Mehnaz Ejaz Khokhar, Nauman Shaukat, Safee Ullah Haider, Absar Ahmed Zafar, Leonardo A Marquez Roa

**Affiliations:** 1 Cardiovascular Medicine, Hayatabad Medical Complex Peshawar, Peshawar, PAK; 2 General Surgery, Cleveland Clinic Foundation, Cleveland, USA; 3 Surgery, Aga Khan University, Karachi, PAK; 4 College of Medicine, Khyber Medical College, Peshawar, PAK; 5 College of Medicine, Dow International Medical College, Karachi, PAK; 6 Cardiovascular Medicine, Royal Cornwall Hospitals NHS Trust, Truro, GBR; 7 Internal Medicine, Shaikh Khalifa Bin Zayed Al-Nahyan Medical and Dental College, Lahore, PAK; 8 Pathology, Cleveland Clinic Foundation, Cleveland, USA; 9 Anesthesiology, Cleveland Clinic Foundation, Cleveland, USA

**Keywords:** cardioimmunology, immune checkpoint inhibitors, immune-mediated myocarditis, inflammation and atherosclerosis, therapeutic immunomodulation

## Abstract

Cardiovascular diseases (CVDs) are the leading cause of death across the world, resulting in a significant number of deaths each year. It has become clear that, alongside traditional risk factors, inflammation and immune dysregulation play a key role in the progression of these diseases. This review explores the new domain where immunology meets cardiology, particularly the bidirectional relationship between immunotherapy and CVDs. Treatments such as immune checkpoint inhibitors (ICIs) and chimeric antigen receptor (CAR) T-cell therapy were originally developed for cancer and autoimmune illnesses. They are currently under evaluation both for their potential applications in CVD and for the cardiotoxic effects associated with their administration. Mechanistic insights from translational research show that a complex interplay between cytokine signalling, autoimmunity, and vascular injury determines both the therapeutic benefit and toxicity. Not only this, but recent evidence has found that the use of anti-inflammatory drugs, such as interleukin-1 (IL-1) inhibitors and colchicine, notably reduces cardiovascular events, marking a shift towards immune-targeted therapies.

However, the immune-mediated life-threatening conditions like myocarditis and vasculitis still exist, necessitating the need for better risk stratification and collaboration between cardiology and oncology. Future research might be directed towards using biomarker-guided monitoring, new classes of immunomodulators, and an overall precision-based approach to achieve both effectiveness and safety. Immunotherapy serves as a promising paradigm shift in cardiovascular medicine, linking two different fields in a way that redefines disease prevention and management.

## Introduction and background

Cardiovascular diseases (CVDs) are the leading cause of death worldwide, accounting for approximately one-third of all global mortality. The World Health Organization (WHO) estimates that annual deaths due to CVDs will reach 23 million by 2030. Prevention and management strategies have primarily targeted traditional risk factors, including hypertension, diabetes, dyslipidemia, smoking, and obesity. However, emerging evidence suggests that immune dysregulation and chronic inflammation play a central role in the development of CVD [1].

Growing evidence also identifies clonal hematopoiesis of indeterminate potential (CHIP) as a significant cardiovascular risk factor [2]. Due to age-related genetic alterations, hematopoietic stem cells in the bone marrow expand disproportionately, leading to heightened inflammatory activity. Increased risks of atherosclerosis, heart failure, aortic valve disease, and ischemic heart disease are associated with CHIP, further emphasizing its role in age-related cardiovascular pathology. Growing evidence also identifies CHIP as a significant cardiovascular risk factor [2]. In addition, biological sex influences immune activation and inflammatory responses, which may modify both susceptibility to CHIP-related CVD and downstream clinical outcomes.

Atherosclerosis, the hallmark of coronary artery disease (CAD), is no longer viewed solely as a lipid storage disorder but rather as a chronic inflammatory disease of the arterial wall [3]. Plaque instability caused by lipid accumulation results in the activation of macrophages, dendritic cells, and T lymphocytes. The collective release of pro-inflammatory cytokines - including interleukin-1β (IL-1β), interleukin-6 (IL-6), and tumor necrosis factor-α (TNF-α) - drives inflammation through multiple mechanisms: IL-1β stimulates the secretion of additional inflammatory mediators; IL-6 enhances B- and T-cell activation, promoting chronic inflammation [4]; and TNF-α initiates a cytokine cascade capable of causing extensive myocardial damage [5].

Autoimmune mechanisms have also been implicated in myocarditis, pericarditis, and certain cardiomyopathies. Autoimmunity-associated cardiovascular risk is driven by shared pathogenic features such as leukocyte dysfunction and elevated cytokine levels, which contribute to vascular dysfunction, oxidative stress, and dysregulated inflammation. Clinically, this is exemplified by accelerated atherosclerosis in rheumatoid arthritis and immune-mediated pericardial involvement in systemic lupus erythematosus. Furthermore, the two primary hallmarks of autoimmunity - loss of self-antigen tolerance and the production of autoantibodies - can also be observed in atherosclerotic heart disease [6].

Emerging research also highlights the gut microbiome as an important regulator of cardiovascular health through its influence on systemic inflammation. Dysbiosis can increase the production of metabolites such as trimethylamine N-oxide (TMAO) and promote the translocation of bacterial components into the circulation, both of which enhance chronic inflammatory signaling [7]. In addition to these mechanisms, modulation of the gut microbiome through dietary interventions, prebiotics, and metabolite-targeted strategies is being explored as a potential therapeutic approach in CVD.

The rapid advancement of immunotherapy has transformed cancer treatment, particularly with the development of immune checkpoint inhibitors (ICIs) and adoptive cell transfer technologies. Early clinical successes with adoptive transfer using tumor-infiltrating lymphocytes demonstrated tumor regression in approximately 75% of patients. Subsequent refinements have incorporated genetic engineering of T cells to express specific T-cell receptors or chimeric antigen receptors (CARs), enabling highly targeted recognition and elimination of malignant cells [8]. Although these innovations have significantly improved cancer outcomes, they have also introduced a range of immune-mediated cardiovascular toxicities, including myocarditis, pericardial disease, arrhythmias, and accelerated atherosclerosis [9,10].

Thus, the relationship between the immune system and the heart is bidirectional: immune activation can both contribute to and help treat CVDs. This concept underpins the expanding field of cardio-immunology, which seeks to identify new immune-based therapeutic targets for CVD while shielding patients from therapy-induced toxicity.

These insights illustrate that CVD is a complex immunoinflammatory condition shaped by environmental factors and immune-tissue interactions. Advances in genomics, immune profiling, and molecular imaging now enable more precise characterization of the individual immune signatures that drive cardiovascular pathology. Cardio-immunology paves the way toward precision cardiovascular medicine guided by personalized immune and molecular profiles rather than traditional risk factors alone. The purpose of this review is to synthesize these emerging developments and delineate the mechanistic and clinical intersections between immunotherapy and CVD. By integrating foundational immunobiology with evolving therapeutic evidence, this review contributes a perspective that is not commonly emphasized in conventional cardiovascular literature, thereby underscoring its relevance and novelty for both research and clinical practice.

## Review

Immunoinflammatory mechanisms driving CVDs

Inflammation plays a dual role in cardiovascular homeostasis. Although it is essential for tissue repair after acute injury, it becomes detrimental when chronically activated. In atherosclerosis, oxidized low-density lipoprotein (oxLDL) acts as a pro-inflammatory ligand that recruits monocytes, which differentiate into macrophages and foam cells, perpetuating plaque growth. Pro-inflammatory cytokines such as IL-1β, TNF-α, and interferon-γ (IFN-γ) further amplify endothelial dysfunction and smooth muscle proliferation [11].

The adaptive immune response further sustains inflammation through the activation of CD4⁺ T-helper cells and B cells that produce autoantibodies against apolipoprotein B (ApoB) and heat shock proteins. These immune responses, in turn, lead to plaque destabilization and, consequently, increase susceptibility to rupture and thrombosis [12].

In myocarditis, viral infection or immune checkpoint inhibition can trigger T-cell-mediated injury to cardiomyocytes, leading to lymphocytic infiltration and myocyte necrosis. Similarly, pericarditis in autoimmune disorders such as systemic lupus erythematosus results from autoantibody production and complement activation, which sustain pericardial inflammation and fibrosis [13].

Innate lymphoid cells, particularly ILC2s, also play an important role in cardiac immune responses. ILC2s contribute to tissue repair after myocardial injury; however, when they remain chronically activated, they can promote cardiac remodeling and fibrosis through sustained type-2 immune signaling. This shift from repair toward fibrosis increases ventricular stiffening and the risk of heart failure progression [14].

In atherosclerosis, activated neutrophils release neutrophil extracellular traps (NETs), which amplify cytokine signaling and destabilize plaques, leading to myocardial infarction (MI), thrombosis, and ischemia [15]. They accelerate plaque progression and increase the risk of acute coronary events. Although NETs themselves are not yet directly targeted in clinical practice, several experimental strategies, such as NET inhibition and enzymatic NET degradation, are under investigation and highlight their potential as future therapeutic targets.

Recent studies have also identified the role of the NLRP3 inflammasome, a type of innate immune sensor that links metabolic stress to heart injury. Activation of NLRP3 induces IL-1β release, a key driver of both atherosclerosis and heart failure with mildly reduced ejection fraction (HFmrEF). Hence, targeting these pathways offers a promising novel therapeutic approach [16].

Cardiovascular research was once focused on lowering lipid levels. However, over the past few years, attention has shifted from lipid-lowering therapies toward interventions that target inflammation. This change began with the CANTOS trial [17]. In this trial, when interleukin-1β (IL-1β) was blocked with canakinumab, the risk of cardiovascular events was significantly reduced, even without any change in lipid levels [18]. In fact, the CANTOS trial demonstrated a 24% reduction in MI, a 10% reduction in cardiovascular death, a 36% reduction in urgent revascularization, and a 37% reduction in cardiac arrest [19].

The COLCOT and LoDoCo2 trials provided further evidence that inflammation-driven cardiovascular risk can be reduced [17,18]. In both studies, low-dose colchicine therapy suppressed neutrophil activation and inhibited inflammasome activity. As a result, the rates of MI and stroke were significantly reduced.

The RHAPSODY trial extended these findings beyond atherosclerosis, showing that rilonacept, an IL-1 inhibitor, is an effective treatment for recurrent pericarditis [20]. Patients who received rilonacept experienced a nearly 74% decrease in disease recurrence. These findings highlight how modulating inflammation can stabilize disease processes previously considered hemodynamic or metabolic in origin. Other IL-1-blocking agents, including anakinra and canakinumab, have shown benefit in inflammatory cardiovascular disorders, supporting the broader relevance of IL-1 pathway inhibition. These therapies are generally compatible with standard cardiac medications, with potential interactions arising mainly from overlapping effects on hemodynamics, renal function, or infection risk rather than direct pharmacologic conflict.

Recent advances in molecular biology have catalyzed the exploration of targeted biologics for treating CVD. For example, inclacumab is a monoclonal antibody that targets P-selectin. It helps lower leukocyte adhesion and reduce heart damage in acute coronary syndromes [21,22]. Researchers are also investigating vaccination strategies against oxLDL and ApoB epitopes, which could offer benefits for atheroprotection. These approaches may provide long-lasting immune tolerance to vascular inflammation [23].

Preclinical models have shown that CAR T-cell therapies, modified to target cardiac fibroblasts, may help reverse myocardial fibrosis, although this remains experimental. Collectively, these innovations suggest that precision immunomodulation could reshape preventive and therapeutic cardiology. Together, these findings underscore the pivotal role of inflammation in CVD and highlight the potential of targeted immunomodulatory strategies to transform prevention and treatment.

Immuno-microbiology and cardiovascular health

Immuno-microbiology is closely correlated with cardiovascular health. Oral microbiota provides a clear example: it contributes to the development of CVD through several pathways, including local and systemic inflammation, activation of immune responses, and cytokine production. Additionally, oral bacteria can enter the bloodstream, and microbial-derived products, such as metabolites (e.g., short-chain fatty acids (SCFAs), TMAO, hydrogen sulfide (H_2_S), nitric oxide (NO), and specific toxins (e.g., lipopolysaccharide (LPS), leukotoxin (LtxA)), can exert systemic effects. Collectively, these processes may promote atherosclerosis, endothelial dysfunction, and other cardiovascular disorders [24].

The gut microbiota influences cardiovascular health by regulating blood vessel function, inflammation, cholesterol, and metabolism. Dysbiosis, an imbalance in gut microbes, is linked to atherosclerosis, hypertension, MI, and heart failure. Microbial metabolites like TMAO, SCFAs, and secondary bile acids affect vascular health, clotting, and lipid metabolism. Gut microbial products activate pattern-recognition receptors such as Toll-like receptors (TLRs) and NOD-like receptors (NLRs), thereby driving chronic inflammation. Targeting the gut microbiota through diet, probiotics, prebiotics, or personalized therapies may help reduce cardiovascular risk [25].

Both oral and gut microbiota interact with the immune system in ways that shape cardiovascular outcomes. Therefore, immuno-microbiome-targeted strategies hold significant potential.

Immunotherapy-related cardiotoxicity in oncology: mechanisms and incidence

Although immunotherapy has transformed cancer treatment, its potential to induce cardiac toxicity remains a significant challenge. ICIs such as nivolumab, pembrolizumab, and ipilimumab block regulatory pathways including PD-1/PD-L1 and CTLA-4. While this enhances cytotoxic T-cell activity against tumors, it can also provoke autoimmune responses that inadvertently target cardiac tissue, resulting in ICI-associated myocarditis. This complication occurs in up to 1% of treated patients and carries a mortality rate exceeding 40% [26,27]. Increasing evidence suggests that ICIs accelerate atherosclerosis by enhancing vascular T-cell activation, leading to rapid plaque progression [28].

Immunotherapy-driven myocarditis is linked to metabolic reprogramming within cardiac cells. In mouse models, activated T cells exhibit mitochondrial dysregulation, particularly affecting glycerolipid metabolism pathways. A key protein, DGKZ, appears to mediate these metabolic changes and may explain why activated T cells initiate inflammation within the myocardium [29].

CAR T-cell therapies, which target tumor-associated antigens such as CD19, represent one of the most innovative immunotherapeutic modalities and are being adopted increasingly across oncology. Their potent immune activation can precipitate cytokine release syndrome (CRS), characterized by surges in IL-6, NO, and other inflammatory mediators that may lead to vasoplegic shock and refractory hypotension. In such cases, hydroxocobalamin may serve as an adjunctive therapy by scavenging excess NO and potentially reducing vasopressor requirements [30]. Recognition of the inflammation-driven cardiovascular effects of CAR T-cell therapy, including myocardial depression, arrhythmias, and hemodynamic instability, is therefore important in contemporary cardiovascular care.

Cancer survivors may also develop radiation-induced heart disease. Immune-mediated injury can result in valvular dysfunction, accelerated CAD, endothelial injury, and valvular fibrosis [31-34]. Traditionally, surgical aortic valve replacement (SAVR) has been the preferred therapy; however, transcatheter aortic valve replacement (TAVR) offers a less inflammatory alternative. Immune activation markers, including granulocyte and lymphocyte responses, are significantly lower following TAVR compared with surgical approaches. These findings highlight the importance of balancing oncologic efficacy with the prevention of immune-mediated cardiovascular complications.

Diagnosis and management

Early detection of immunotherapy-related cardiac injury is essential. Elevated troponin and N-terminal prohormone of brain natriuretic peptide (NT-proBNP) levels serve as sensitive but nonspecific markers. Cardiac MRI and endomyocardial biopsy remain the diagnostic gold standards. Emerging biomarkers, such as soluble PD-1 and circulating T-cell signatures, show promise for early risk stratification [35].

High-dose corticosteroids represent first-line therapy. However, steroid-refractory cases require alternative immunosuppressants such as mycophenolate mofetil or abatacept (a CTLA-4 agonist). Multidisciplinary cardio-oncology clinics are increasingly important for guiding complex management and long-term monitoring [36].

Detection of elevated inflammatory biomarkers, including cytokines, troponin, and NT-proBNP, helps clinicians identify immune pathways driving cardiovascular risk. Targeted anti-inflammatory therapy via IL-1 inhibition has emerged as a promising strategy. Agents such as anakinra, rilonacept, and canakinumab suppress systemic inflammation, stabilize atherosclerotic plaques, reduce myocardial injury, and improve overall cardiovascular outcomes, aligning with precision immunomodulation approaches [37,38].

For myocardial injury, particularly MI, microRNAs and extracellular vesicles (EVs) have gained attention as emerging biomarkers. MicroRNAs, including miR-146a, miR-21, and miR-155, regulate inflammatory and immune pathways, and their elevation serves as an indicator of myocardial stress. Endothelial cells, cardiomyocytes, and activated immune cells release EVs containing inflammatory proteins. MicroRNAs are typically quantified using quantitative reverse transcription polymerase chain reaction (qRT-PCR) or next-generation sequencing (NGS). EVs are isolated through ultracentrifugation-based methods and characterized using nanoparticle tracking analysis (NTA) [39]. However, microRNAs lack absolute disease specificity and can be influenced by systemic inflammation and non-cardiac conditions, which limits their diagnostic precision compared with established markers such as troponins.

Positron emission tomography (PET) and echocardiography are integral to the diagnosis and management of cardiovascular inflammation. PET imaging detects metabolic activity, such as increased fluorodeoxyglucose (FDG) uptake, which enables identification of inflammation before structural abnormalities arise [40]. Myocardial contrast echocardiography (MCE) assesses myocardial perfusion and microvascular integrity using microbubble contrast agents; perfusion deficits may indicate microvascular dysfunction associated with inflammation. Contrast-enhanced cardiac MRI (CE-MRI) provides high-resolution tissue characterization and detects myocardial edema, fibrosis, and regional wall-motion abnormalities [41]. When combined with emerging precision-medicine tools, including multi-omics profiling and AI-based risk stratification, these imaging modalities can contribute to a more individualized assessment of inflammatory pathways and disease trajectories. Together, these advanced modalities allow earlier and more precise characterization of cardiovascular inflammation, supporting targeted therapeutic strategies.

Clinical case insights and translational lessons

Clinical case studies have demonstrated the practical impact of immune modulation across cardiovascular settings. Cold agglutinin disease, an IgM-mediated autoimmune hemolytic anemia, creates unique perioperative challenges, particularly in cases involving hypothermia. In such scenarios, pre-treatment with rituximab and maintaining normothermia have proven effective [42,43].

Lifestyle factors can also influence inflammatory pathways, including in exercise-induced recurrent pericarditis. This condition involves repeated episodes of pericardial inflammation that may be exacerbated by strenuous physical activity, which can upregulate IL-1-mediated inflammatory responses. Targeted IL-1 inhibition, particularly when combined with colchicine, has demonstrated durable remission [44].

After cardiac surgery, some patients develop persistent vasoplegic shock, often driven by immune system activation. Such cases respond well to hydroxocobalamin, which can reduce the need for vasopressor medications by up to 70% [45]. These examples illustrate how understanding immune mechanisms supports tailored immunomodulation to improve outcomes in diverse cardiovascular conditions.

Recent advances in managing recurrent pericarditis are supported by meta-analyses and clinical case data emphasizing personalized immunomodulation. Colchicine and IL-1 inhibitors show superior efficacy in preventing recurrences, with fewer adverse events compared to corticosteroids alone, reinforcing the rationale for biologic therapy in difficult-to-treat or recurrent disease [46].

Real-world reports further highlight these principles. For instance, a young athlete with post-cardiac injury syndrome pericarditis was successfully treated with rilonacept, enabling a safe return to high-intensity exercise while limiting inflammatory flares. Additionally, severe vasoplegia after cardiopulmonary bypass, often intensified by cytokine storms, has been effectively managed with hydroxocobalamin, stabilizing hemodynamics and reducing vasopressor dependence in immune-driven shock. These developments underscore the expanding role of immunotherapy in cardio-oncology and perioperative care. Guided by biomarkers, future strategies are expected to become increasingly precise and effective.

Challenges, knowledge gaps, and ethical considerations

Despite promising advances, several major challenges remain. Diagnosing ICI-associated myocarditis lacks standardized criteria, and endomyocardial biopsy continues to be the only definitive diagnostic tool. Long-term cardiovascular effects, such as accelerated atherosclerosis and endothelial dysfunction, remain insufficiently studied. Importantly, emerging evidence suggests that women experience higher rates of ICI-associated myocarditis and exhibit distinct inflammatory and immune-response profiles, underscoring a critical knowledge gap in sex-specific risk assessment [26].

Economic and ethical issues also persist. Biologic immunotherapies are costly, limiting accessibility in low- and middle-income countries. Trials such as CANTOS have demonstrated an increased infection risk associated with systemic immunosuppression. Furthermore, genetic and epigenetic predictors of cardiotoxicity, including HLA polymorphisms, require further investigation to enable personalized approaches.

Long-term monitoring is another major gap. Most cardio-oncology registries emphasize short-term outcomes, highlighting the need for large-scale, prospective studies that incorporate immune, genomic, and imaging biomarkers. While PET offers diagnostic potential, it is hindered by high false-positive rates and limited sensitivity. FDG uptake varies across tissues and can create inconsistent signals; previous use of antibiotics or corticosteroids may further obscure inflammation [47]. Echocardiography also has limitations, particularly in congenital heart disease, where certain structural abnormalities may be missed.

Overcoming cardiotoxicity associated with ICIs is a central challenge for expanding their therapeutic applications [48]. Although cytokine-based therapies show early promise, dose-limiting toxicity and poor pharmacokinetics hinder their broader use. Similarly, CAR T-cell therapy, despite its oncologic efficacy, can induce cardiovascular complications, particularly in patients with high tumor burden or severe CRS [49]. These challenges emphasize the need for refined and targeted immunotherapies that maximize therapeutic benefit while minimizing cardiovascular risk.

Limitations

This review has several limitations that should be acknowledged. First, it is a narrative review rather than a systematic review, and therefore does not follow a predefined search strategy or formal quality assessment of included studies. As a result, selection bias cannot be fully excluded. Second, much of the available evidence on immunotherapy-related cardiovascular effects is derived from short- to medium-term follow-up, and the long-term cardiovascular consequences of immune modulation, including accelerated atherosclerosis and chronic endothelial dysfunction, remain incompletely characterized. Third, cost and accessibility barriers limit the real-world applicability of biologic immunotherapies, particularly in low- and middle-income countries, raising important equity considerations. Finally, there is a lack of head-to-head clinical trials directly comparing different immunomodulatory agents, which restricts conclusions regarding relative efficacy and safety across therapies. These limitations highlight the need for large, prospective studies with longer follow-up and more comprehensive comparative designs.

Future directions: toward precision cardio-immunology

Precision medicine in cardio-immunology is emerging, with molecular signatures increasingly being used to direct therapy and predict toxicity. Artificial intelligence (AI) is also being applied to evaluate individualized risk based on immunologic and imaging data. AI models can combine multi-omics data with imaging-based inflammation scores and basic clinical factors. They can then guide IL-1 inhibitor use, support ICI therapy surveillance, and help monitor fibrosis-targeted CAR T-cell treatments. Multidisciplinary cardio-oncology teams and global collaborations will be pivotal in harmonizing protocols and ensuring the safe use of immunotherapy in patients with underlying heart disease.

Other strategies under investigation include modified CAR T-cell therapy for fibrotic heart disease, atherosclerosis-related vaccines, next-generation cytokine inhibitors with improved safety profiles, and combination strategies such as prophylactic administration of statins or aspirin in patients receiving ICIs to reduce the risk of immune-related atherosclerosis.

With the growing demand for more comprehensive imaging, hybrid techniques have begun to reshape clinical diagnostics. PET is increasingly expected to become a valuable tool through hybrid PET/MRI models, which bridge molecular and systemic diagnosis and facilitate functional and precise treatment planning [50].

Nanoparticle-based delivery systems offer a precise and personalized approach. Nanotechnology provides an alternative to the limitations of cytokine-based therapy, including dose-limiting toxicity and suboptimal pharmacokinetics [51]. Combining antibodies targeting IL-1β and TNF-α with molecular-weight polysaccharides results in highly localized antibody activity while mitigating systemic side effects [52].

Emerging tools for precision immunomodulation

In the future, immunotherapy will increasingly focus on designing immune interventions and customized treatment for each inflammatory context. One such innovation is single-cell profiling, enabling the identification of patient-specific therapeutic targets by characterizing pathogenic cellular patterns in inflammatory lesions. Since conventional histological and imaging methods are unreliable in distinguishing responders from non-responders, single-cell profiling has become essential. Multimodal single-cell assays link gene expression, chromatin states, and clonal T-cell dynamics, enabling a better understanding of pathogenic behavior. Single-cell RNA sequencing (scRNA-seq) reveals the heterogeneity of cell populations within atherosclerotic plaques, myocarditis lesions, and post-infarction remodeling zones [53]. Furthermore, single-cell profiling may analyze microbiota-derived metabolites, microbial composition, and host immune responses to identify novel interaction pathways [54].

Spatial transcriptomics also enhances immunomodulation by mapping gene expression across anatomic regions while preserving immune cell architecture. It is highly beneficial for atherosclerosis: it identifies plaques enriched with inflammatory macrophages, enabling early detection [55,56].

Real-time immunodiagnostics, NGS, and mass spectrometry also enable advanced cell profiling [57]. A new type of mass spectrometry, multiplex proteomics, can quantify proteins across multiple samples in a single experiment. Multi-omics approaches integrate data from different omic levels, enhancing the functional analysis of human disease. When integrated with machine learning, these approaches may transform precision-based immunomodulation [58].

## Conclusions

Immunotherapy has transformed modern medicine and blurred the boundaries between oncology, immunology, and cardiology. Although immune modulation provides a powerful approach against chronic inflammation and atherosclerosis, it also introduces unprecedented cardiovascular risks. Balancing efficacy with safety requires integrated care models, robust biomarker discovery, and continued translational research. The field of cardio-immunology thus holds the potential to shift cardiovascular medicine from reactive management to preventive, personalized, and immune-guided care.
